# Ulipristal Acetate Modifies miRNA Expression in Both Superficial and Basal Layers of the Human Endometrium

**DOI:** 10.3390/jcm10194442

**Published:** 2021-09-27

**Authors:** Kamila Kolanska, Maria Sbeih, Geoffroy Canlorbe, Arsène Mekinian, Justine Varinot, Perrine Capmas, Martin Koskas, Selim Aractingi, Emile Daraï, Nathalie Chabbert-Buffet

**Affiliations:** 1 INSERM UMRS 938, Sorbonne Université, Site Saint-Antoine, 27 rue Chaligny, CEDEX 12, 75571 Paris, France; maria_sbeih@hotmail.com (M.S.); geoffroy.canlorbe@aphp.fr (G.C.); martin.koskas@aphp.fr (M.K.); selim.aractingi@aphp.fr (S.A.); emile.darai@aphp.fr (E.D.); nathalie.chabbert-buffet@aphp.fr (N.C.-B.); 2 Service de Gynécologie Sestertius et Médecine de la Reproduction, AP-HP Sorbonne Université Site Tenon, 4 rue de la Chine, 75020 Paris, France; 3 Department of Gynecological and Breast Surgery and Oncology, Pitié-Salpêtrière University Hospital, Assistance Publique des Hôpitaux de Paris (AP-HP), 75013 Paris, France; 4 Service de Médecine Interne, AP-HP Sorbonne Université Site St Antoine, 184 rue du Faubourg Saint Antoine, 75012 Paris, France; arsene.mekinian@aphp.fr; 5 Service d’Anatomopathologie, AP HP Sorbonne Université Site Tenon, 4 rue de la Chine, 75020 Paris, France; justine.varinot@aphp.fr; 6 Department of Gynecology and Obstetrics, University Paris Saclay, 78 rue du Général Leclerc, 94270 Le Kremlin-Bicêtre, France; perrine.capmas@aphp.fr; 7 Inserm, Centre of Research in Epidemiology and Population Health (CESP), U1018, 94276 Le Kremlin-Bicêtre, France; 8 Department of Obstetrics and Gynecology, AP-HP Bichat University Hospital, 75018 Paris, France; 9 Institut de Recherche en Santé de la Femme, Equipe d’accueil 7285, Universite de Versailles Saint-Quentin-en-Yvelines, 78180 Montigny-le-Bretonneux, France

**Keywords:** ulipristal acetate, endometrium, miRNA, basal and functional layer

## Abstract

(1) Background: Ulipristal acetate (UPA) is a selective progesterone receptor modulator (SPRM) widely used for emergency contraception and mid- to long-term leiomyoma treatment. The aim of this study was to identify modifications of miRNA expression in superficial and basal layers of the human endometrium at the end of the UPA treatment for at least 3 months. (2) Methods: Microarray miRNA analysis of formalin-fixed, paraffin-embedded hysterectomy tissue samples was conducted, followed by an Ingenuity Pathway Analysis. Samples were divided into three groups: women having had 3 months of UPA treatment (*n* = 7); and two control groups of UPA-naïve women in the proliferative (*n* = 8) or secretory (*n* = 6) phase. (3) Results: The UPA modified the expression of 59 miRNAs involved in the processes of cell cycle, carcinogenesis, and inflammation. Their expression profiles were different in the basal and superficial layers. Most of the processes influenced by the UPA in the basal layer were connected to the cell cycle and immune regulation. (4) Conclusion: Specific changes were observed in both layers of the endometrium in the UPA group. However, the miRNA expression in the basal layer was not consistent with that in the superficial layer. Other large studies analysing the long-term impact of SPRM on endometrial miRNA expression are necessary.

## 1. Introduction

Ulipristal acetate (UPA) is a selective progesterone receptor modulator (SPRM). It binds to the major progesterone receptor (PR) isoforms and exerts a wide spectrum of actions, from antagonistic to agonistic effects, depending on the target tissue [1]. It is widely used for emergency contraception and, in some countries, as repetitive 3-month cures in leiomyoma treatment [2]. New indications are currently being developed for women desiring to conceive [3,4].

UPA, like other SPRMs, has a reversible impact on the morphological aspect of the whole endometrium [5]. Endometrial thickening is observed on ultrasound scan in 10% of UPA treated patients, and a specific histological aspect called PR modulator-associated endometrial changes (PAEC) [6,7] has also been observed in up to 59% of endometrial biopsies at treatment week 13 with a subsequent decrease to 8% after treatment discontinuation [8].

At a molecular level in the myoma tissue, UPA has been shown to modify a number of processes including a decrease in inflammation and induction of cell apoptosis [9,10]. Specific modifications of PR and progesterone-regulated markers of endometrial maturation expression in endometrial biopsies under the action of UPA have been recently described by Whitaker et al. [11]. The authors also report modifications in the expression patterns of the corresponding mRNA. Furthermore, the expression of the genes responsible for endometrial receptivity has been reported to be modified by mid-cycle administration of UPA [12]. However, little is known about the long-term endometrial effects of UPA, and the molecular mechanisms of mRNA and protein expression regulation have not been fully elucidated.

MicroRNA (miRNA) are small non-coding RNAs. They modify the expression of around 60% of proteins at the posttranscriptional level and promote mRNA destruction. The miRNAs are involved in a broad variety of physiological mechanisms such as morphogenesis, differentiation, apoptosis or cellular metabolism [13].

Some miRNAs have been associated with endometrial receptivity [14] and fluctuate according to progesterone blood levels during controlled ovarian hyperstimulation [15].

There are no data about the miRNA expression regulation in UPA exposed endometrium. Furthermore, comparing miRNA expression in the superficial and basal layers of the endometrium could provide information about the potential persistence of UPA impact on the endometrium. In fact, the superficial layer sheds every month while the basal layer persists.

The aim of this study was to identify modifications of miRNA expression, and thus the potential target pathways, in both superficial and basal layers of the human endometrium after 3 months of UPA treatment, before treatment discontinuation, in women treated for symptomatic myomas.

## 2. Materials and Methods

### 2.1. Sample Collection

Endometrium-containing blocks of hysterectomy samples from non-menopausal women aged 40–52 years with moderate to severe symptoms of myomas requiring hysterectomy between 2014 and 2017 were retrospectively collected in three university hospital surgical gynaecology units in Paris, France (Tenon Hospital, Bichât Hospital and Kremlin–Bicêtre Hospital). Women who had hormonal treatment for at least 3 months before surgery or a history of cancer were excluded. The clinical variables of the women included age, parity and body mass index (BMI, calculated as weight in kilograms divided by the square of height in meters). The localisation of the myoma according to the FIGO classification [16] and with regard to the analysed endometrial specimen location has been noted (Table S1 in Supplementary Materials). The samples included in the UPA group were obtained from women operated on after a 3-month course of UPA, before treatment discontinuation.

The menstrual phase was assessed on a histological basis and on evaluation of the ER and PR expressions [17,18]. The presence of progesterone receptor modulator associated endometrial changes (PAEC) were specifically assessed [7].

The Obstetrics and Gynecology Research Ethics Committee (CEROG—Comité d’Ethique de la Recherche en Obstétrique et Gynécologie) allowed retrospective sample analysis and approved the study (Approval N° b-4-15). All women gave pre-operative written informed consent for the participation in the study.

### 2.2. Endometrial Dating

Formalin-fixed, paraffin-embedded (FFPE) tissue sample sections of 3 μm were processed and stained with Haematoxylin and Eosin by standard methods. 

Immunohistochemistry of the ER and PR were performed on the Ventana Ultra platform using the manufacturer’s recommended settings.

Whole tissue sections (4-µm) were deparaffinized, rehydrated, treated and incubated for 16 min with the pre-diluted anti-ER antibody (monoclonal rabbit anti-human ER clone SP1, Roche Diagnostics, Roche, Meylan, France) and the pre-diluted anti-PR antibody (monoclonal rabbit anti-human PR clone 1E2, Roche Diagnostics, Roche, Meylan, France), and then visualized using the ultraView Universal DAB Detection Kit (Roche Diagnostics, Roche, Meylan, France).

All evaluations were performed under blinded conditions by a single expert pathologist (CB). Endometrial differentiation was assessed by histological dating according to the Noyes criteria [17], and oestrogen receptor (ER) and PR expression [18] were analysed by the expert pathologist as previously described [19]. 

### 2.3. RNA Extraction from FFPE Tissues, Microarray Hybridization and DATA Analysis

FFPE tissues were obtained from hysterectomy specimens. Macro-dissections were undertaken with a surgical scalpel on 20 μm sections under a light microscope to separate the superficial and the basal layers of the endometrium. The superficial layer sampling included the superficial endometrial epithelium and the first row of endometrial glands and scattered stroma. The basal layer sampling included the juxta-myometrial portion of the endometrium with dense stroma. The intermediate layer of the endometrium was excluded from analysis. The samples were stored at 4 °C and RNA extraction was performed within 2 h.

Total RNA was extracted using the miRNeasy FFPE Kit (Qiagen, Courtaboeuf, France) according to the manufacturer’s instructions. The purity, quality and concentration of all RNA samples were examined by using a microspectrophotometer Bioanalyzer 2100 (Agilent Technologies Inc., Waldbronn, Germany) [20]. The analysis was conducted at the genomic platform of the Institut Cochin, Paris.

Microarray analysis was conducted on seven samples from seven women treated for at least 3 months with UPA and who were still using it (UPA group), eight samples from eight control women in the proliferative phase (proliferative phase group) and six samples from six women in the secretory phase (secretory phase group). Microarray hybridization on the miRNA 4.0 chips (Affymetrix^®^, Santa Clara, CA, USA) was conducted at the genomic platform of the Institut Cochin, Paris. Each sample was tested once as the microarray hybridization is a robust technology with a broadband analysis. Specific miRNA analysis was performed using Partek^®^ Flow^®^ software version 3.0 (Partek Inc., St. Louis, MO, USA). CEL files were imported and normalized using robust multi-array averaging (RMA) [21]. Those miRNA with a nominal *p*-value ≤ 0.05 were considered to be differentially expressed. The miRNA showing a 2-fold change or more were considered for further analysis. The results were synthetically presented using Venn diagrams. 

Evaluation of the impact of UPA on bio-functions and canonical pathways associated with endometrial receptivity was conducted using Ingenuity Pathway Analysis software (IPA: http://www.ingenuity.com, accessed on 30 April 2018).

### 2.4. Statistical Analysis

Unless otherwise specified, data were managed using an Excel database and analysed using R version 3.1.3 (R Foundation for Statistical Computing), available online at https://www.r-project.org/ (accessed on 19 March 2018).

## 3. Results

### 3.1. Population and Endometrium Characteristics

**Figure 1:**
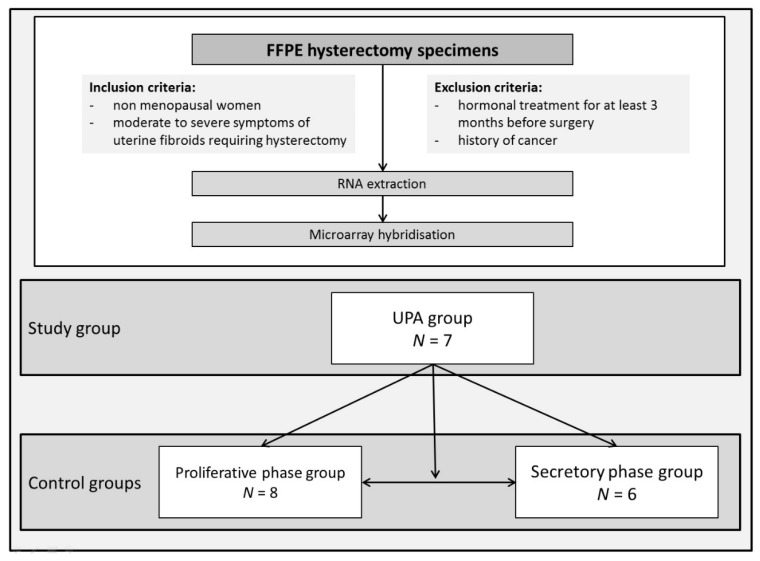
Flow chart. RNA was extracted from FFPE hysterectomy specimens from 21 non-menopausal women presenting moderate to severe symptoms of myoma requiring hysterectomy. The study group included 7 women operated on after a 3-month course of UPA, before treatment discontinuation. Two control groups consisted of 8 hysterectomy samples obtained in the proliferative phase and 6 hysterectomy samples obtained in the secretory phase. FFPE: Formalin-fixed, paraffin-embedded; UPA: Ulipristal acetate.

Three groups of patients were included in the study. The UPA group included women operated on after a 3-month course of UPA, before treatment discontinuation (Esmya, GEDEON RICHTER FRANCE, 5 mg daily) (*n* = 7) (Figure 1). Two distinct control groups included samples obtained from UPA-naïve women undergoing a hysterectomy for symptomatic myoma in the proliferative phase (proliferative phase group, *n* = 8) or the secretory phase (secretory phase group, *n* = 6). The menstrual phase was assessed on a histological basis and on ER and PR expression evaluations [17,18].

The baseline characteristics of the three study groups—the UPA group, and the proliferative phase and secretory phase groups—were comparable (Table S1). The FIGO classification of myomas and their localisation to the proximity of the analysed endometrium was not statistically different between the study groups (Table S1). The minimum distance between the myoma and endometrial sample was 5 mm.

In the UPA group, focal and not pronounced PAEC were observed in two cases (29%). The proliferative aspect of the endometrium in the UPA group was observed in six samples and one endometrium was characterised by an early secretory aspect.

### 3.2. Effect of UPA on Global miRNA Expression in Endometrial Samples (GEO: GSE150231)

Endometrial miRNA profiles in the UPA, proliferative phase and secretory phase groups were analysed in each endometrial layer separately using microarray hybridization.

The median ratio of the RNA integrity number (RIN) of the analysed samples was 2.3 (interquartile range (IQR) 2.2–2.4) and the median concentration of RNA was 66.0 ng/μL (IQR 36.5–102.8).

**Figure 2:**
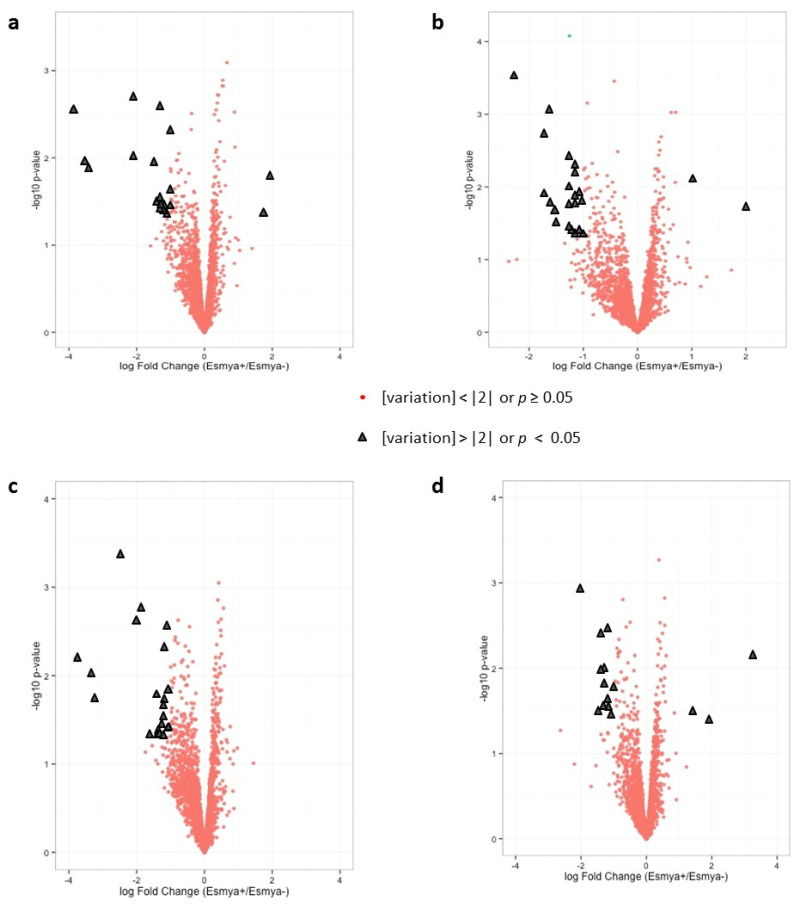
Volcano plot. Abscissa is the logarithmic value of the level of variation (log-ratio) and ordinate is the negative logarithm of the statistical value (−log (*p*)) of fluorescence intensities of the hybridized probes. The cut-off of *p* < 0.05 (−log *p* > 1.3) and fold change (FC) > |2| identified two up-regulated and 17 down-regulated miRNA in UPA group versus proliferative phase group in superficial layer (**a**); two up-regulated and 17 down-regulated miRNA in UPA group versus secretory phase group in superficial layer (**b**); 21 down-regulated miRNA in UPA group versus proliferative phase group in basal layer (**c**); and three up-regulated and 12 down-regulated miRNA in UPA group versus secretory phase group in basal layer (**d**).

The expression of miRNA was differentially regulated in the UPA group as compared to the proliferative and secretory phase groups in both the basal and superficial layers of the endometrium (Figure 2 and Table 1).

In the superficial layer the cut-off values for *p* < 0.05 and fold change > |2| identified two up-regulated and 17 down-regulated miRNAs in the UPA group compared to the proliferative phase group. According to the IPA analysis they were experimentally proven to regulate three and 269 mRNAs, respectively. 

**Figure 3:**
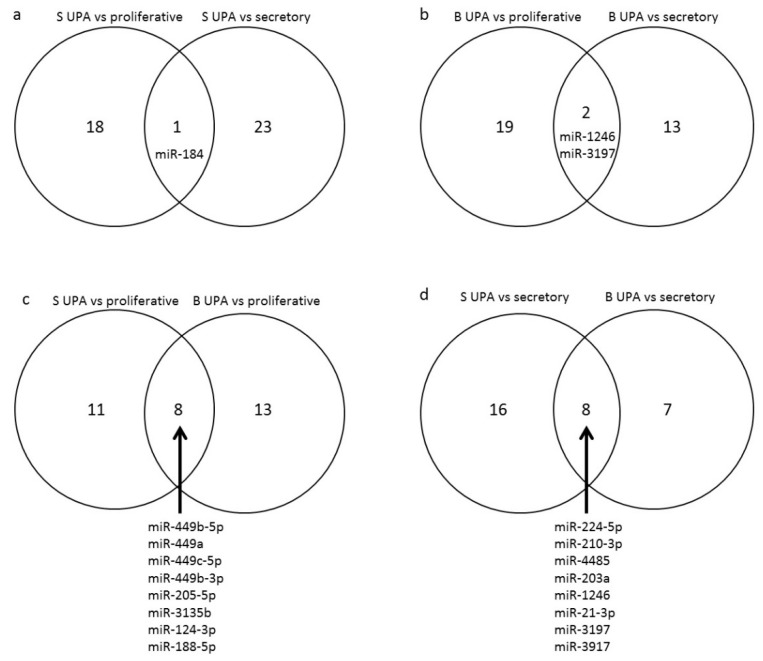
Venn diagrams representing common miRNA identified in the analyses. In the superficial layer (S), 18 miRNA were differentially expressed in UPA group as compared to the proliferative phase group and 23 as compared to secretory phase group. miR-184 was differentially expressed in the UPA group as compared to both the proliferative and secretory phase groups (**a**). In the basal layer (B), 19 miRNA were differentially expressed in the UPA group as compared to the proliferative phase group and 13 miRNA as compared to the secretory phase group. miR-1246 and miR-3197 were differentially expressed in the UPA group compared to both the proliferative and secretory phase groups (**b**). When comparing UPA to the proliferative phase group, 11 miRNA were differentially expressed in the superficial layer, 13 in the basal layer and 8 miRNA (miR-449b-5p, miR-449a, miR-449c-5p, miR-449b-3p, miR-205-5p, miR-3135b, miR-124-3p, miR-188-5p) were differentially expressed in both endometrial layers (**c**). When comparing UPA to the secretory phase group, 16 miRNA were differentially expressed in the superficial layer, 7 in the basal layer and 8 (miR-224-5p, miR-210-3p, miR-4485, miR-203a, miR-1246, miR-21-3p, miR-3197, miR-3917) were differentially expressed in both endometrial layers (**d**).

Two miRNAs were up-regulated and 22 down-regulated in the UPA group when compared to the secretory phase group. They were experimentally proven to regulate 23 and 125 mRNAs, respectively. Among these miRNAs, miR-184 was differentially expressed in the UPA group as compared to both the proliferative and secretory phase groups (Figure 3a).

In the basal layer, 21 miRNAs were down-regulated and none up-regulated in the UPA group as compared to the proliferative phase group. They were experimentally proven to regulate 444 mRNAs. In comparison to the secretory phase group, three miRNAs were up-regulated and 12 down-regulated in the UPA group. These miRNAs regulate the expression of 11 and 103 mRNA, respectively. Among these miRNAs, miR-1246 and miR-3197 were differentially expressed in the UPA group compared to both the proliferative and secretory phase groups (Figure 3b). 

Among the 59 miRNAs identified as being modified by the UPA treatment, only 16 showed the same UPA-related profile in both layers of the endometrium. Among these, eight miRNAs were differentially expressed in the UPA group as compared to the proliferative phase group (miR-449b-5p, miR-449a, miR-449c-5p, miR-449b-3p, miR-205-5p, miR-3135b, miR-124-3p, miR-188-5p) (Figure 3c) and eight were differentially expressed in the UPA group as compared to the secretory phase group (miR-224-5p, miR-210-3p, miR-4485, miR-203a, miR-1246, miR-21-3p, miR-3197, miR-3917) (Figure 3d).

### 3.3. Functional Analyses

#### 3.3.1. Gene Ontology Analysis

Using IPA, we conducted a functional analysis of the mRNAs regulated by the miRNA previously described in the miRNA expression section. The top 10 statistically most significant biological processes potentially involved in the physiopathology of the human endometrium for each analysis group are presented in Table S2. Processes most significantly modulated by UPA were connected to the cell cycle, apoptosis, tumorigenesis, and inflammatory process.

#### 3.3.2. Modified State of Canonical Pathways

IPA analysis of the list of mRNAs of interest (i.e., those regulated by the miRNAs differentially expressed in UPA-treated cycles) identified the top ten canonical pathways involved. The results are presented in Table S3.

The most significantly influenced canonical pathways were connected to the cell cycle, cell signalling linked to cancer development and inflammation. The miRNAs involved in these processes were down-regulated.

## 4. Discussion

The aim of this study was to analyse the effect of the UPA on the endometrial expression of miRNA as compared to the proliferative and secretory phases in superficial and basal layers of the human endometrium. The analysis revealed 59 miRNAs differentially expressed in the UPA group. The identified miRNAs showed differential expression profiles in the basal and superficial layers.

Among the identified miRNAs, miR-449a, miR-449b-3p, miR-449b-5p and miR-449c-5p showed the biggest fold change in both endometrial layers. They were down-regulated in the UPA group as compared to the proliferative phase group. These miRNAs are known to be up-regulated in the endometrium of women with repeated embryo implantation failure (RIF) during the implantation window [22]. The miR-30b-5p, miR-1246, miR-1973 and miR-4485, which are also reported to be up-regulated during the implantation window in the endometrium of women with RIF [22,23], were down-regulated in the endometrium exposed to UPA as compared to the secretory phase control group. The miR-196a-5p, up-regulated in the implantation window of women with RIF [22], was found to be up-regulated in the UPA group as compared to the secretory phase group. Three miRNAs (miR-21-3p, miR-188-5p and miR-205-5p) have been reported to be down-regulated during the implantation window in women with RIF [23]. In this study, the miR-21-3p was found to be down-regulated under UPA as compared to the secretory phase. Both miR-188-5p and miR-205-5p were found to be down-regulated under UPA as compared to the proliferative phase control group. 

Among the identified miRNAs, miR-188-5p has been described to be up-regulated in endometrial cancer as compared to normal endometrium [24]. In our study, its expression was down-regulated in the UPA group as compared to the proliferative phase group in both endometrial layers, which suggests that the UPA does not have a pro-tumoral action. This has been confirmed in various clinical studies showing the absence of UPA associated endometrial carcinoma and the regression of the specific endometrial changes during and after treatment cessation. miR-429 has been described as being up-regulated in the superficial layer of women with endometrial cancer as compared to normal endometrium samples [25]. In our study, it was up-regulated by UPA treatment as compared to the secretory phase in the superficial layer and unchanged as compared to the proliferative phase. As endometrial dating is not provided in the endometrial cancer study [25] a comparison with our results is hindered. The miR-28-5p, miR-663b and miR-339-5p were shown to be up-regulated in endometrial cancer compared to normal endometrium [25] whereas in our study they were down-regulated by UPA treatment as compared to the secretory phase for miR-28-5p and the proliferative phase for miR-663b and miR-339-5p. miR-542-5p was found to be down-regulated both in endometrial cancer [25] and by the UPA as compared to the proliferative phase in the superficial layer.

The literature data on the involvement of identified miRNA in embryo implantation and endometrial cancer are resumed in Table 2 [26].

Our IPA analysis of the top ten statistically most significant biological processes regulated by the identified miRNA showed that UPA might potentially indirectly modify the expression of ESR1 (ERα) and PTEN mRNA. The expression of these mRNAs has previously been found unchanged in endometrium exposed to UPA [11]. However, mRNA expression is regulated by a complex network of molecular interactions which is obviously not limited to miRNA action. 

UPA binds to both major isoforms of progesterone receptors and, together with transcription co-regulators, induces or inhibits the signal transduction [1,27]. Its endometrial impact is characterised by progesterone agonist and antagonist actions. Some UPA specific effects can also be observed. Both miR-1246 and miR-3197 are specifically modulated as compared to the proliferative and secretory phases in both endometrial layers. The miR-1246 has been described as up-regulated during the implantation window of women with RIF [23]. This suggests a specific mixed agonist and antagonist effect of UPA.

The impact of UPA on the basal layer of the endometrium suggests a potential long-term impact since the superficial functional layer sheds with menstrual bleeding, while the basal layer persists. The endometrial basal layer may be considered as the ‘memory’ of the endometrium, as it could be a source of regeneration of the functional layer among others mechanisms, by harboring endometrial stem cells [28]. However, at the molecular level, it has been shown that fragments of the shed menstrual superficial layer were enriched in mRNAs associated with extracellular matrix synthesis [29], suggesting that the endometrium regenerates from fragments of the superficial layer rather than from the basalis. Several reports showed a differential expression of mRNAs between the superficial layer and basal layer of the endometrium, reflecting their specific differentiation. However, such data are not available for miRNA. In our study, some changes in miRNA expression in the basal layer could be observed, which are not consistent with the miRNA expression changes in the superficial layer. In addition, in our previous work that focused on protein expression [19], a discrepancy was also observed between basalis and functionalis layers, reinforcing the hypothesis of functional differences between the layers. Therefore, the impact of UPA may be different in the different endometrial layers, but our results do not allow predictions on the long-term effect of UPA on implantation. In fact, by assessing only women who had their surgery performed before stopping the use of UPA, the impact of menstrual shedding on what was observed cannot be assessed. Further studies would be necessary to confirm the long-term effect of UPA on the endometrium. The miRNA expression in the superficial layer of the endometrium obtained by endometrial biopsies at different times from the UPA treatment disruption could help to prove our results.

The presence of PAEC in two out of seven samples was observed. This ratio is higher than that described in the literature. However, the changes were focal and not pronounced.

The major strength of our study was the large number of patients drawn from a homogeneous population of women with symptomatic myomas included in the analysis. Furthermore, as we analysed hysterectomy samples, we were able to evaluate both endometrial layers. The miRNA analysis was performed using a robust technology with a broadband analysis. The quality control was undertaken for all samples before the analysis.

The limitations of this study included its retrospective design and the absence of hormonal assessment of the menstrual cycle phase. However, as symptomatic myomas usually require hormonal treatment, the number of control samples was limited. Another limitation was that all the included patients were experiencing severe myoma-related symptoms and our data cannot be extrapolated to other clinical situations. UPA licence has been discontinued since March 2020 due to severe adverse events and more particularly to liver cell damage, therefore larger studies on UPA in the indication of uterine leiomyoma are impossible in the current circumstances. However, the data may be of scientific interest for other SPRM molecules with mixed agonist and antagonist effects. Finally, the use of paraffin-embedded samples reduced the quality of total RNA (RIN around two). RIN is poorly informative on the miRNA quality obtained from FFPE tissue according to the literature [30,31] and may underestimate miRNA quality since miRNAs are rather stable RNAs because of their double-stranded structure and so artefacts due to RNA degradation in these low RIN samples may have hampered our results [32]. Further studies on frozen samples would be necessary.

## 5. Conclusions

Our results show that UPA specifically modifies the expression of 59 miRNAs, the majority of which have been described as being involved in processes connected to embryo implantation and cancerogenesis. The changes in miRNA expression observed in the basal layer, from which the functional layer renews every month, are different from the molecular changes observed in the superficial layer.

While miRNA can act as promoters or inhibitors of cancerogenesis, the regulation of miRNA observed in the UPA exposed endometria was opposite to the modifications described in the literature for tumoral endometria in all but two miRNA. Other large studies analysing the role of miRNAs in the endometrium and the long-term impact of SPRMs on endometrial miRNA expression are necessary.

miRNAs are involved in the inflammatory processes playing essential roles in embryo implantation. All but four miRNA were oppositely expressed in the UPA exposed endometrium compared to the published data on the endometria of RIF women.

These results deserve further confirmation but suggest that UPA might be used in women willing to conceive without impairing the chances of pregnancy or increasing the risks of developing endometrial malignancies.

## Figures and Tables

**Table 1:** List of up- or down-regulated miRNA in the UPA group as compared to the proliferative or secretory phase groups in each layer of the human endometrium.

			miRNA	*p*	FC	Number of Experimentally Proven * Regulated mRNA
Superficial layer	Different from proliferative	Up-regulated	hsa-miR-184	0.016	3.79	3
			hsa-miR-3613-5p	0.044	3.42	
		Down-regulated	hsa-miR-449b-5p ^2^	0.003	−14.97	269
			hsa-miR-449a ^1^	0.011	−11.72	
			hsa-miR-449c-5p ^3^	0.013	−10.83	
			hsa-miR-449b-3p ^4^	0.010	−4.42	
			hsa-miR-205-5p ^5^	0.002	−4.40	
			hsa-miR-3135b ^6^	0.011	−2.85	
			hsa-miR-362-5p	0.032	−2.59	
			hsa-miR-370-3p	0.029	−2.57	
			hsa-miR-124-3p ^7^	0.038	−2.51	
			hsa-miR-542-5p	0.033	−2.50	
			hsa-miR-663b	0.003	−2.47	
			hsa-miR-188-5p ^8^	0.039	−2.41	
			hsa-miR-339-5p	0.034	−2.29	
			hsa-miR-1244	0.045	−2.14	
			hsa-miR-6728-5p	0.005	−2.04	
			hsa-miR-193b-3p	0.023	−2.01	
			hsa-miR-501-3p	0.036	−2.00	
	Different from secretory	Up-regulated	hsa-miR-184	0.020	3.99	23
			hsa-miR-429	0.008	2.02	
		Down-regulated	hsa-miR-224-5p ^9^	0.0003	−4.93	125
			hsa-miR-210-3p ^10^	0.002	−3.34	
			hsa-miR-1298-3p	0.013	−3.32	
			hsa-miR-4485 ^11^	0.001	−3.12	
			hsa-miR-203a ^12^	0.017	−3.08	
			hsa-miR-30b-5p	0.022	−2.91	
			hsa-miR-1247-5p	0.032	−2.89	
			hsa-miR-28-5p	0.038	−2.41	
			hsa-miR-4655-5p	0.004	−2.41	
			hsa-miR-1246 ^13^	0.018	−2.40	
			hsa-miR-4428	0.010	−2.40	
			hsa-miR-3163	0.000	−2.39	
			hsa-miR-21-3p ^14^	0.041	−2.36	
			hsa-miR-1973	0.044	−2.27	
			hsa-miR-6718-5p	0.013	−2.25	
			hsa-miR-4667-5p	0.017	−2.25	
			hsa-miR-30d-5p	0.006	−2.22	
			hsa-miR-4513	0.005	−2.20	
			hsa-miR-4685-5p	0.012	−2.12	
			hsa-miR-3197 ^15^	0.042	−2.11	
			hsa-miR-3917 ^16^	0.044	−2.04	
			hsa-miR-7846-3p	0.015	−2.02	
Basal layer	Different from proliferative	Down-regulated	hsa-miR-449c-5p ^3^	0.006	−14.00	444
			hsa-miR-449b-5p ^2^	0.009	−10.13	
			hsa-miR-449a ^1^	0.018	−9.72	
			hsa-miR-205-5p ^5^	0.0004	−5.59	
			hsa-miR-124-3p ^7^	0.002	−4.01	
			hsa-miR-3135b ^6^	0.002	−3.69	
			hsa-miR-449b-3p ^4^	0.047	−3.09	
			hsa-miR-887-3p	0.016	−2.67	
			hsa-miR-15a-5p	0.047	−2.59	
			hsa-miR-378f	0.042	−2.55	
			hsa-miR-378d	0.046	−2.46	
			hsa-miR-188-5p ^8^	0.037	−2.44	
			hsa-miR-7162-3p	0.022	−2.36	
			hsa-miR-500b-3p	0.030	−2.30	
			hsa-miR-125b-2-3p	0.049	−2.26	
			hsa-miR-4788	0.005	−2.25	
			hsa-miR-1246 ^13^	0.019	−2.23	
			hsa-miR-3907	0.003	−2.16	
			hsa-miR-6824-5p	0.039	−2.10	
			hsa-miR-6875-5p	0.014	−2.07	
			hsa-miR-3197 ^15^	0.038	−2.04	
	Different from secretory	Up-regulated	hsa-miR-196a-5p	0.0074	9.37	11
			hsa-miR-3613-5p	0.0429	3.78	
			hsa-miR-615-3p	0.0327	2.60	
		Down-regulated	hsa-miR-224-5p ^9^	0.0012	−4.10	103
			hsa-miR-203a ^12^	0.0325	−2.73	
			hsa-miR-210-3p ^10^	0.0108	−2.66	
			hsa-miR-4485 ^11^	0.0039	−2.65	
			hsa-miR-21-3p ^14^	0.0282	−2.53	
			hsa-miR-146b-5p	0.0156	−2.52	
			hsa-miR-3917 ^16^	0.0102	−2.51	
			hsa-miR-1246 ^13^	0.0247	−2.29	
			hsa-miR-3911	0.0034	−2.28	
			hsa-miR-3197 ^15^	0.0291	−2.24	
			hsa-miR-7111-5p	0.0357	−2.06	
			hsa-miR-4669	0.0169	−2.01	

UPA: ulipristal acetate; FC: fold-change. 1–16: miRNA with modified expression in the superficial and basal layers. * direct association between miRNA expression and mRNA regulation previously published, analysis done using Ingenuity Pathway Analysis (IPA: http://www.ingenuity.com, accessed on 30 April 2018).

**Table 2:** Literature data on the involvement of identified miRNAs in embryo implantation and endometrial cancer.

	UPA Action on miRNA	Embryo Implantation	Endometrial Cancer
miR-21-3p	down-regulated compared to secretory phase	down-regulated in RIF during implantation window [23]	
miR-28-5p	down-regulated compared to secretory phase		up-regulated in endometrial cancer [25]
miR-30b-5p	down-regulated compared to secretory phase	up-regulated in RIF during implantation window [22,23]	
miR-188-5p	down-regulated compared to proliferative phase	down-regulated in RIF during implantation window [23]	up-regulated in endometrial cancer compared to normal endometrium [24]
miR-196a-5p	up-regulated compared to secretory phase	up-regulated in RIF during implantation window [22]	
miR-205-5p	down-regulated compared to proliferative phase	down-regulated in RIF during implantation window [23]	
miR-339-5p	down-regulated compared to proliferative phase		up-regulated in endometrial cancer compared to normal endometrium [25]
miR-429	up-regulated compared to secretory phase		up-regulated in endometrial cancer compared to normal endometrium samples [25]
miR-449a	down-regulated compared to proliferative phase	up-regulated in RIF during implantation window [22]	
miR-449b-3p	down-regulated compared to proliferative phase	up-regulated in RIF during implantation window [22]	
miR-449b-5p	down-regulated compared to proliferative phase	up-regulated in RIF during implantation window [22]	
miR-542-5p	down-regulated compared to proliferative phase		down-regulated in endometrial cancer compared to normal endometrium [25]
miR-663b	down-regulated compared to proliferative phase		up-regulated in endometrial cancer compared to normal endometrium [25]
miR-449c-5p	down-regulated compared to proliferative phase	up-regulated in RIF during implantation window [22]	
miR-1246	down-regulated compared to secretory phase	up-regulated in RIF during implantation window [22,23]	
miR-1973	down-regulated compared to secretory phase	up-regulated in RIF during implantation window [23,24]	
miR-4485	down-regulated compared to secretory phase	up-regulated in RIF during implantation window [22,23]	

## Data Availability

Availability of data and material: GEO. Code availability: GSE150231.

## Supplementary Materials

The following are available online at https://www.mdpi.com/article/10.3390/jcm10194442/s1, Table S1: Population and endometrial characteristics, Table S2: selection of the top ten statistically most significant biological processes potentially involved in the physiopathology of human endometrium, Table S3: selection of the top ten canonical pathways regulated by the miRNA identified in Table 1.
