# Supplementary material for: Ulipristal Acetate Modifies miRNA Expression in Both Superficial and Basal Layers of the Human Endometrium
Source: J Clin Med. 2021 Sep 27;10(19):4442. doi: 10.3390/jcm10194442 (PMC8509688; doi:10.3390/jcm10194442)
Supplement: Supplementary file 1 [file jcm-10-04442-s001.zip › jcm-1388909-supplementary.pdf]

Table S1. Population and endometrial characteristics.

.	Proliferative Phase Group (n = 8)	Secretory Phase Group (n = 6)	UPA Group (n = 7)	p
Age (years) median (IQR)	47.5 (45.3-50.5)	47.0 (46.0-49.3)	48.0 (45.0-50.0)	0.95 [1]
BMI kg/m² mean (CI95%)	27.2 (23.2-31.2)	27.9 (22.1-33.7)	28.0 (21.9-34.0)	0.96 [2]
Tobacco smoking *n* (%)	0 (0)	2 (40)	2 (29)	0.25 [3]
Parity *n* (%)				0.27 [3]
0	1 (12.5)	2 (33.3)	1 (14.3)	
1	1 (12.5)	2 (33.3)	4 (57.1)	
> 1	6 (75.0)	2 (33.3)	2 (28.6)	
FIGO classification				0.57 [3]
1-2	1	3	2	
3-5	6	3	4	
6-7	0	0	0	
NA	1	0	1	
Myoma neighbouring to the analysed endometrium	3	3	2	0.73 [3]
Presence of focal PAEC	NA	NA	2	NA

[1] Non-parametric Kruskal Wallis; [2] ANOVA test variance analysis.[3] Chi² test; IQR – interquartile range; CI95% – 95% confidence interval.

Table S2. Selection of the top ten statistically most significant biological processes potentially involved in the physiopathology of human endometrium.

		Biological Processes	-log(p-value)	mRNA Participating in the Selected Biological Processes and Regulated by Identified miRNA
Superficial layer Different from proliferative	Up-regulated	Quantity of connective tissue cells	5.47	AKT2,NFATC2,SFRP4
		Cell death of connective tissue cells	4.29	AKT2,NFATC2,SFRP4
		Migration of cancer cells	3.59	AKT2,NFATC2
		Metastasis of tumour cell lines	3.32	AKT2,NFATC2
		Remission of tumour	3.09	AKT2
		Transmigration of cancer cells	3.01	NFATC2
		Apoptosis of fibroblast cell lines	3.00	AKT2,NFATC2
		Binding of leukocytes	2.93	AKT2,NFATC2
		Migration of cells	2.89	AKT2,NFATC2,SFRP4
		Binding of DNA	2.85	AKT2,NFATC2
	Down	Transcription of RNA	21.22	AHR,AHRR,AR,BCL6,BDNF,BTG3,CAV1,CCL2,CCND1,CDK2,CDK6,CEBPA,CREB1,CREB3L2,CTGF,DLL1,DLX2,E2F3,E2F5,E2F6,EGR1,ELF4,ELK3,ESR1,ETS1,FOXA2,GMCL1,GSK3B,HDAC1,HDAC4,HD

			AC5,HES1,HOXA1,HOXA11,INPPL1,JAG1,KLF15,LDLR,MAP2K1,MAPK14,MDFIC,MED1,MET,MYB,MYC,MYCN,NEUROD1,NFATC1,NFIC,NOTCH1,NOTCH2,PHF19,PPP1R13L,PRKCE,PRKD1,PTBP1,PTEN,RARG,RELA,SIRT1,SMAD5,SNAI2,SOX9,SP1,STAT3,TEAD1,TOM1L1,TP53,TRIM29,TRIP11,TRPS1,TWIST2,UHRF1,WNT1,ZBED3,ZEB1,ZEB2
	G1/S phase transition	20.50	AHR,BCL2,CCND1,CDK2,CDK4,CDK6,CEBPA,E2F3,E2F5,ESR1,ITGB1,MAP2K1,MAPK14,MET,MYB,MYC,NFATC1,PTEN,PTK2,STAT3,TP53,VEGFA,WNT1,ZEB2
	Interphase	20.32	AHR,ARAF,BCL2,CAV1,CCND1,CDK2,CDK4,CDK6,CEBPA,E2F3,E2F5,E2F6,EGR1,ESR1,HDAC1,HDAC4,ITGB1,MAD2L2,MAP2K1,MAP3K8,MAPK14,MET,MYB,MYC,MYCN,NEK9,NFATC1,NOTCH1,PTEN,PTK2,RELA,SIRT1,SOX9,STAT3,TP53,UHRF1,VEGFA,WNT1,ZEB2
	Transcription	19.98	AHR,AHRR,AR,BCL6,BDNF,BTG3,CAV1,**CCL2**,CCND1,CDK2,CDK6,CEBPA,CREB1,CREB3L2,CTGF,DLL1,DLX2,E2F3,E2F5,E2F6,EGR1,ELF4,ELK3,ERBB3,ESR1,ETS1,FOXA2,GAS2L1,GMCL1,GSK3B,HDAC1,HDAC4,HDAC5,HES1,HOXA1,HOXA11,INPPL1,JAG1,KLF15,LDLR,MAP2K1,MAPK14,MDFIC,MED1,MET,MYB,MYC,MYCN,NEUROD1,NFATC1,NFIC,NOTCH1,NOTCH2,PHF19,PPP1R13L,PRKCE,PRKD1,PTBP1,PTEN,RARG,RELA,SIRT1,SMAD5,SNAI2,SOX9,SP1,STAT3,TEAD1,TOM1L1,TP53,TRIM29,TRIP11,TRPS1,TWIST2,UHRF1,VEGFA,WNT1,ZBED3,ZEB1,ZEB2
	Differentiation of epithelial tissue	19.65	ABHD5,AR,BCL2,BLOC1S6,CAV1,CCND1,CEBPA,CTNND1,DLL1,F11R,FOXA2,HDAC1,HES1,ITGB1,JAG1,MAP2K1,MAPK14,MET,MYB,MYC,MYH9,NEUROD1,NFATC1,NOTCH1,NOTCH2,PLAU,PPP1R13L,PTEN,RDH10,RELA,SOX9,STAT3,TOM1L1,VEGFA,ZEB1,ZEB2
	Apoptosis	19.17	AHR,AR,AXIN2,B4GALT1,BCL2,BCL6,BDNF,CAV1,**CCL2**,CCND1,CD164,CD47,CD59,CDK2,CDK4,CDK6,CEBPA,CERS2,CREB1,CREB3L2,CTGF,CYP1B1,DEPDC1,DFFB,DHCR24,DLL1,DLX2,DRAM1,E2F3,EGR1,ELF4,ERBB3,ESR1,ETS1,FOXA2,GMCL1,GSK3B,GSN,HDAC1,HDAC4,HDAC5,HEBP2,HES1,HOXA11,INPPL1,ITGB1,JAG1,LDLR,MAD2L2,MAN2A1,MAP2K1,MAP3K8,MAPK14,MCL1,MED1,MET,MTPN,MYB,MYC,MYCN,NEUROD1,NFATC1,NFIC,NOTCH1,NOTCH2,PGF,PLAU,PLP2,PLSCR3,PPP1R13L,PRKCE,PTEN,PTK2,RARG,RASSF5,RELA,SIRT1,SLC17A5,SMAD5,SNAI2,SOX9,SP1,STAT3,SWAP70,SYCP1,TEAD1,TJP2,TMEM109,TNFRSF21,TP53,TRIM29,TRPS1,VEGFA,WNT1,ZEB2,ZFP36L2
	Expression of RNA	18.90	AHR,AHRR,AR,BCL6,BDNF,BTG3,CAV1,CCL2,CCND1,CDK2,CDK6,CEBPA,CREB1,CREB3L2,CTGF,DLL1,DLX2,DNAJC1,E2F3,E2F5,E2F6,EGR1,ELF4,ELK3,ERBB3,ESR1,ETS1,FOXA2,GAS2L1,GMCL1,GSK3B,HDAC1,HDAC4,HDAC5,HES1,HOXA1,HOXA11,INPPL1,JAG1,KLF15,LDLR,MAP2K1,MAPK14,MDFIC,MED1,MET,MYB,MYC,MYCN,NEUROD1,NFATC1,NFIC,NOTCH1,NOTCH2,PHF19,PPP1R13L,PRKCE,PRKD1,PTBP1,PTEN,RARG,RELA,SIRT1,SMAD5,SNAI2,SOX9,SP1,STAT3,TEAD1,TOM1L1,TP53,TRIM29,TRIP11,TRPS1,TWIST2,UHRF1,VEGFA,WNT1,ZBED3,ZEB1,ZEB2
	Cell transformation	18.86	ARAF,ARHGEF1,BCL2,BCL6,CAV1,CCND1,CDK2,CDK4,CDK6,CEBPA,DFFB,EGR1,ERBB3,ESR1,ETS1,HES1,HTATIP2,ITGB1,MAP2K1,MAP3K8,MAPK14,MET,MYB,MYC,MYCN,NFATC1,NOTCH1,PPP1R13L,PRKCE,PTEN,PTK2,PTPN12,RELA,RHOG,SOX9,STAT3,TP53,

				VEGFA,WNT1
		Cell cycle progression	18.22	AHR,ARAF,ARPC1B,B4GALT1,BCL2,BCL6,BTG3,CAV1,CCND1,CD59,CDK2,CDK4,CDK6,CEBPA,CREB1,DLX2,E2F3,E2F5,E2F6,EGR1,ERBB3,ESR1,ETS1,GAS2L1,GSK3B,HDAC1,ITGB1,MAD2L2,MAP2K1,MAPK14,MET,MYB,MYC,MYCN,NEK6,NEK9,NEUROD1,NFATC1,NOTCH1,PTEN,PTK2,RARG,RELA,SIRT1,STAT3,TP53,UHMK1,VEGFA,WNT1,ZEB1
		Solid tumour	17.61	AHR,ALDH9A1,AR,ARAF,BCL2,BCL6,BDNF,BTG3,CAV1,**CCL2**,CCND1,CD47,CDCA7L,CDK2,CDK4,CDK6,CEBPA,CREB1,CTGF,CTNND1,CYP1B1,DLL1,DNM2,DVL2,E2F3,EGR1,ERBB3,ESR1,ETS1,FAM129B,FAM57A,GSK3B,HDAC1,HTATIP2,IQGAP1,ITGB1,MAP2K1,MAP3K8,MAPK14,MCL1,MET,MYB,MYC,MYCN,MYH9,NEUROD1,NFATC1,NOTCH1,NOTCH2,PGF,PGM1,PLAU,PPP1R13L,PRKCE,PTBP1,PTEN,PTK2,RASSF5,RELA,RHOG,SIRT1,SNAI2,STAT3,STX2,TLN1,TNFRSF21,TP53,TRIM29,UHRF1,VEGFA,WNT1,ZEB1,ZEB2
Different from secretory	Up-regulated	Endometrial cancer	6.90	BAP1,ERRFI1,PTEN
		Quantity of cells	5.90	AKT2,ERBIN,ERRFI1,NFATC2,PLCG1,PTEN,PTPN12,PTPN13,RERE,SFRP4,ZEB1,ZEB2
		Migration of cells	5.80	AKT2,ELMO2,ERRFI1,NFATC2,PLCG1,PTEN,PTPN12,RERE,SFRP4,ZEB1,ZEB2
		Endometrial adenocarcinoma	5.48	BAP1,PTEN
		Metastasis of cells	4.80	AKT2,NFATC2,PTEN,ZEB1,ZEB2
		Hyperplasia of endometrium	4.51	ERRFI1,PTEN
		Migration of cancer cells	4.50	AKT2,NFATC2,PTEN,ZEB1
		Solid tumour	4.46	AKT2,BAP1,ERBIN,ERRFI1,NFATC2,PTEN,PTPN13,ZEB1,ZEB2
		Differentiation of epithelial tissue	4.45	ERBIN,ERRFI1,PTEN,ZEB1,ZEB2
		Neoplasia of tumour cell lines	4.40	AKT2,BAP1,NFATC2,PTEN,ZEB2
	Down-regulated	Solid tumour	11.58	ABL1,ANPEP,ATP2A2,BCL6,BECN1,CDCP1,CTGF,CXCL14,E2F3,F2,GALNT1,GNAI2,IDH1,IL11,ITGA2,JUN,MAP4K4,MBNL1,MET,MLLT1,MNT,NEUROD1,NPTX1,NT5E,PGM1,PTPN1,RAD52,RUNX2,SDHD,SLC7A11,SLC9A3R2,SMAD4,SOCS3,SRC,STX1A,TNRC6A,TP53,TP63,WNT5A
		Malignant solid tumour	10.36	ABL1,ANPEP,ATP2A2,BCL6,BECN1,CTGF,CXCL14,E2F3,F2,GNAI2,IL11,ITGA2,JUN,MAP4K4,MBNL1,MET,MLLT1,NEUROD1,NPTX1,NT5E,PGM1,PTPN1,RAD52,RUNX2,SLC7A11,SMAD4,SOCS3,SRC,TNRC6A,TP53,TP63,WNT5A
		Cancer	10.01	ABL1,ANPEP,ATP2A2,BCL6,BECN1,CBFB,CDCP1,CTGF,CXCL14,E2F3,F2,GNAI2,HNRNPM,HOXA1,IL11,ISCU,ITGA2,JUN,MAP4K4,MBNL1,MET,MLLT1,NEUROD1,NPTX1,NT5E,PGM1,PNP,PTPN1,RAD52,RUNX2,SLC7A11,SMAD4,SOCS3,SRC,TNRC6A,TP53,TP63,UBE2I,WNT5A
		Cell viability	7.49	ABL1,BCL6,BECN1,CBFB,CTGF,E2F3,EFNA3,IL11,JUN,MET,MNT,NEUROD1,PPP3CA,PTPN1,RAD52,RUNX2,SMAD4,SOCS3,SRC,STX1A,TP53,TP63,WNT5A
		Tumorigenesis of malignant tumour	7.28	ABL1,BCL6,BECN1,GNAI2,JUN,MBNL1,MET,NEUROD1,RUNX2,SLC7A11,SMAD4,SOCS3,TNRC6A,TP53,TP63,WNT5A

	Apoptosis	7.17	ABL1,ANPEP,API5,ATRX,BCL6,BECN1,CASP8AP2,CBFB,CDCP1,CTGF,E2F3,F2,FGFRL1,GNAI2,IL11,JUN,MAP4K4,MET,MNT,NCEH1,NCL,NEUROD1,PAFAH1B2,PEX11B,PNP,PPP3CA,PTPN1,RUNX2,SLC4A7,SLC7A11,SMAD4,SOCS3,SRC,STX1A,TNFAIP2,TNRC6A,TP53,TP63,TRPS1,UBE2I,WNT5A
	Connective tissue tumour	6.76	ABL1,BECN1,IDH1,JUN,MAP4K4,MET,MLLT1,NT5E,RUNX2,SLC7A11,TP53,TP63
	Differentiation of tumour cell lines	6.52	ABL1,ACVR1,BCL6,CNOT9,IL11,JUN,NEUROD1,SMAD4,SOCS3,TP53
	Proliferation of connective tissue cells	6.46	ABL1,CTGF,F2,GNAI2,IDH1,JUN,MET,NPR3,PTPN1,RUNX2,SLC7A11,SMAD4,SOCS3,SRC,TP53,TP63,TRPS1,WNT5A
	Connective tissue cancer	6.39	JUN,MET,NT5E,RUNX2,TP53,TP63
Basal layer Different from proliferative Down-regulated	Interphase	26.35	AHR,ARAF,BCL2,CAV1,CCND1,CCND3,CCNE1,CDC25A,CDK2,CDK4,CDK6,CEBPA,CENPJ,CHEK1,DMTF1,E2F3,E2F5,EGR1,EIF4E,FGF2,FLT3,GRB10,GRB2,HDAC1,HDAC4,IGF1,IGF1R,ITGB1,JUN,KITLG,MAD2L2,MAP2K1,MAPK14,MAPK3,MET,MSH2,MYB,MYC,MYCN,NEK9,NFATC1,NOTCH1,PTEN,PTGS2,PURA,RAF1,RELA,SIRT1,SOX9,SPI1,STAT3,TP53,UHRF1,VEGFA,WNT1,ZEB2
	Cell transformation	25.93	ARAF,ARHGEF1,BCL2,CAV1,CCND1,CCND3,CCNE1,CDC25A,CDK2,CDK4,CDK6,CEBPA,DFFB,DMTF1,EGFR,EGR1,EIF4E,ERBB3,F2,FGF2,FGFR1,FLT3,GOLGA5,GRB10,GRB2,HES1,HMGA1,HTATIP2,IGF1,IGF1R,ITGB1,JUN,MAP2K1,MAP2K4,MAPK14,MET,MLLT1,MYB,MYC,MYCN,NFATC1,NOTCH1,ODC1,PDCD4,PISD,PPP1R13L,PRKCE,PTEN,PTPN12,RAF1,RELA,RHOG,SOX9,STAT3,TP53,TPM3,VEGFA,WNT1
	Cell cycle progression	25.59	AHR,ARAF,ARPC1B,B4GALT1,BCL2,BTG3,CAV1,CCND1,CCND3,CCNE1,CCNF,CD59,CDC25A,CDK2,CDK4,CDK6,CEBPA,CENPJ,CHEK1,CREB1,DLX2,DMTF1,E2F3,E2F5,EGFR,EGR1,ERBB3,F2,FGF2,FGF7,FGFR1,GAS2L1,GRB10,GRB2,GSK3B,GTF2H1,HDAC1,IGF1,IGF1R,ITGB1,JUN,KITLG,MAD2L2,MAP2K1,MAP2K4,MAPK14,MAPK3,MET,MSH2,MYB,MYC,MYCN,NEK6,NEK9,NEUROD1,NFATC1,NOTCH1,PLAG1,PLK1,PRIMPOL,PTEN,PTGS2,PURA,RAF1,RARG,RELA,SIRT1,STAT3,TP53,UBE2S,UHMK1,VEGFA,WEE1,WNT1,WNT3A,ZEB1
	Malignant solid tumour	25.51	AHR,ALDH9A1,ARHGDIA,ASXL2,BCL2,BTG3,CAV1,**CCL2**,CCND1,CCND3,CD47,CDCA7L,CDK2,CDK4,CDK6,CEBPA,CHEK1,CREB1,CTGF,CTNND1,CYP1B1,DMTF1,E2F3,EGFR,EGR1,ERBB3,F2,FGF2,FGF7,FGFR1,FNDC3B,GRB2,HACE1,HDAC1,HMGA1,HMOX1,HSP90B1,HTATIP2,IGF1,IGF1R,IQGAP1,ITGA2,ITGB1,JUN,KITLG,LAMTOR5,MAP2K1,MAPK14,MCL1,MET,MLLT1,MSH2,MYB,MYC,MYCN,MYH9,NEUROD1,NFATC1,NFIA,NOTCH1,NOTCH2,ODC1,PDCD4,PGF,PGM1,PLK1,PRKCE,PTBP1,PTEN,PTGS2,RAD51C,RAF1,RASSF5,RECK,RELA,RHOG,SIRT1,SKAP2,SNAI2,SPI1,SQSTM1,STAT3,STX2,TLN1,TNFRSF21,TP53,TPI1,TRIM29,TUSC2,UHRF1,VEGFA,WNT1,WNT3A,WT1,ZEB1,ZEB2,ZYX
	Solid tumour	24.46	AHR,ALDH9A1,ARAF,ARHGDIA,ASXL2,BCL2,BDNF,BTG3,CAV1,CCL2,CCND1,CCND3,CCNE1,CD47,CDC25A,CDCA7L,CDK2,CDK4,CDK6,CEBPA,CHEK1,CREB1,CSHL1,CTGF,CTNND1,CYP1B1,DLL1,DMTF1,DNM2,DVL2,E2F3,EGFR,EGR1,EIF4E,ERBB3,F2,FAM129B,FAM57A,FGF2,FGF7,FGFR1,FNDC3B,GRB2,GSK3B,HACE1,HDAC1,

		HMGA1,HMOX1,HSP90B1,HTATIP2,IGF1,IGF1R,IQGAP1,ITGA2,ITGB1,JUN,KITLG,LAMTOR5,MAP2K1,MAP2K4,MAPK14,MCL1,MET,MLLT1,MSH2,MYB,MYC,MYCN,MYH9,NEUROD1,NFATC1,NFIA,NOTCH1,NOTCH2,ODC1,PDCD4,PGF,PGM1,PISD,PLK1,PPP1R13L,PRKCE,PTBP1,PTEN,PTGS2,RAD51C,RAF1,RASSF5,RECK,RELA,RHOG,SIRT1,SKAP2,SNAI2,SPI1,SQSTM1,STAT3,STX2,TLN1,TNFRSF21,TP53,TPI1,TRIM29,TUSC2,UHRF1,VEGFA,WNT1,WNT3A,WT1,ZEB1,ZEB2,ZYX
Apoptosis	22.41	AHR,ATF6,AXIN2,B4GALT1,BCL2,BCL2L2,BDNF,CADM1,CAPRIN1,CAV1,**CCL2**,CCND1,CCND3,CCNE1,CD164,CD47,CD59,CDC25A,CDK2,CDK4,CDK6,CEBPA,CENPJ,CERS2,CHEK1,CREB1,CREB3L2,CREBL2,CTGF,CYP1B1,DEPDC1,DFFB,DHCR24,DLL1,DLX2,DRAM1,E2F3,EGFR,EGR1,EIF4E,ELF4,ERBB3,F2,FGF2,FGFR1,FLT3,FOXA2,GFPT1,GMCL1,GPAM,GRB10,GSK3B,GSN,HDAC1,HDAC4,HDAC5,HEBP2,HES1,HMGA1,HMOX1,IGF1,IGF1R,IGF2R,INPPL1,ITGB1,JAG1,JUN,KITLG,LDLR,MAD2L2,MAN2A1,MAP2K1,MAP2K4,MAPK14,MAPK3,MCL1,MED1,MET,MSH2,MTPN,MYB,MYC,MYCN,NEUROD1,NFATC1,NFIC,NOTCH1,NOTCH2,ODC1,OGT,OMA1,PAFAH1B2,PANX1,PDCD4,PDCD6IP,PGF,PLK1,PLP2,PLSCR3,PNP,PPP1R13L,PRKCE,PTEN,PTGS2,RAF1,RARG,RASSF5,RELA,RNASEL,RTN4,SERPINE2,SIRT1,SLC17A5,SMAD5,SNAI2,SOX9,SP1,SPI1,SQSTM1,STAT3,SWAP70,SYCP1,TEAD1,TIA1,TJP2,TMEM109,TNFRSF21,TP53,TRIM29,TRPS1,TXN2,UCP2,VEGFA,VTI1B,WEE1,WNT1,WNT3A,WT1,ZEB2,ZFP36L2
Tumorigenesis of tissue	22.29	AHR,ARAF,BCL2,BDNF,CAV1,CCL2,CCND1,CCND3,CCNE1,CDK2,CDK4,CDK6,CEBPA,CHORDC1,CREB1,CSHL1,CTGF,CTNND1,DLL1,E2F3,EGFR,EGR1,ERBB3,FAM57A,FGF2,FGF7,FGFR1,FLT3,GRB2,GSK3B,HMGA1,HMOX1,HTATIP2,IGF1,IGF1R,IQGAP1,ITGA2,ITGB1,JUN,LAMTOR5,MAP2K1,MAP2K4,MET,MSH2,MYC,MYCN,NFATC1,NOTCH1,NOTCH2,ODC1,PDCD4,PGF,PISD,PLK1,PRKCE,PTEN,PTGS2,RAD51C,RAF1,RASSF5,RECK,RELA,RHOG,SIRT1,SNAI2,SPI1,STAT3,STX2,TP53,TPI1,TRIM29,TUSC2,VEGFA,WNT1,WNT3A,WT1
Proliferation of fibroblast cell lines	21.87	AHR,ARHGEF1,ATG9A,BTG3,CAPRIN1,CAV1,CCND1,CDC14B,CDK4,CDK6,CEBPA,CREB1,DMTF1,E2F3,EGFR,EGR1,EIF4E,ERBB3,F2,FGF2,GRB2,HMGA1,IGF1,IGF1R,ITGB1,JAG1,JUN,LDLRAP1,MAD2L2,MAPK14,MAPK3,MET,MYC,NFATC1,NOTCH1,ODC1,PLAG1,PPIF,PRKCE,PTEN,RAF1,RASSF5,RECK,RELA,RNASEL,RYK,SIRT1,SOX9,STAT3,SWAP70,TP53,TPM3,WNT3A,WT1
Cell survival	21.62	AHR,BCL2,BCL2L2,BDNF,CADM1,CAV1,**CCL2**,CD47,CD59,CDCA7L,CDK4,CHEK1,CREB1,CTGF,DLX2,E2F3,EGFR,EGR1,EIF4E,ELF4,ERBB3,FGF2,FGF7,FGFR1,FLT3,GRB10,GRB2,GSK3B,HDAC1,HDAC5,HES1,HMGA1,HMOX1,IGF1,IGF1R,IQGAP1,ITGB1,JAG1,JUN,KITLG,MAD2L2,MAP2K1,MAP2K4,MAPK14,MAPK3,MCL1,MET,MSH2,MYB,MYC,MYCN,NEUROD1,NOTCH1,NOTCH2,ODC1,OGT,PDCD4,PGF,PLK1,PPP1R13L,PRKCE,PRKD1,PTEN,PTGS2,RAD51C,RAF1,RARG,RELA,RNASEL,SIRT1,SNAI2,SOX9,STAT3,TP53,UCP2,UHRF1,VEGFA,WIPF1,WNT1,WT1,ZEB1,ZEB2
Cancer	21.30	AHR,ALDH9A1,ARHGDIA,ASXL2,BCL2,BTG3,CAV1,CCL2,CCND1,CCND3,CD47,CD59,CDC25A,CDCA7L,CDK2,CDK4,CDK6,CEBPA,CHEK1,CREB1,CSHL1,CTGF,CTNND1,CYP1B1,DFFB,DLX2,DMTF1,

				E2F3,EGFR,EGR1,ERBB3,F2,FGF2,FGF7,FGFR1,FLT3,FNDC3B,FOXA2,GRB2,GSK3B,GSN,HACE1,HDAC1,HES1,HMGA1,HMOX1,HSP90B1,HTATIP2,IGF1,IGF1R,IGF2R,IQGAP1,ITGA2,ITGB1,JUN,KITLG,LAMTOR5,LITAF,MAD2L2,MAP2K1,MAP2K4,MAPK14,MCL1,MET,MLLT1,MSH2,MYB,MYC,MYCN,MYH9,NEUROD1,NFATC1,NFIA,NOTCH1,NOTCH2,ODC1,PDCD4,PGF,PGM1,PLK1,PNP,PPP1R13L,PRKCE,PTBP1,PTEN,PTGS2,RAD51C,RAF1,RARG,RASSF5,RECK,RELA,RHOG,SIRT1,SKAP2,SNAI2,SPI1,SQSTM1,STAT3,STX2,TLN1,TNFRSF21,TP53,TPI1,TRIM29,TUSC2,UHRF1,VEGFA,WNT1,WNT3A,WT1,ZEB1,ZEB2,ZYX
Different from secretory	Up-regulated	Expression of RNA	5.67	AR,HOXA7,HOXB8,HOXC8,HOXD8,IKBKB,LCOR,S100A9
		Transcription of RNA	4.96	AR,HOXA7,HOXB8,HOXC8,HOXD8,IKBKB,LCOR
		Activation of DNA endogenous promoter	4.90	AR,HOXA7,HOXB8,HOXC8,HOXD8,LCOR
		Regeneration of epithelial tissue	4.48	AR,IKBKB
		Cell death of cancer cells	4.35	ANXA1,AR,HOXA7,IKBKB
		Fibrosis of secretory structure	4.32	AR,IKBKB
		Migration of tumour cell lines	4.10	ANXA1,IKBKB,S100A9
		Morphology of tumour	3.86	ANXA1,AR,IKBKB
		Chemotaxis	3.83	ANXA1,HOXA7,IKBKB,S100A9
		Growth of tumour	3.64	ANXA1,AR,HOXA7,IKBKB,S100A9
	Down-regulated	Inflammation of organ	17.98	ABL1,BLMH,CAMP,CD1D,CD40,CFH,CHUK,CRP,DMBT1,E2F3,IFNB1,IL10,IL11,IL12RB2,IL1R1,IL36RN,IRAK1,IRF5,LBP,LTB,MNT,MR1,NOS2,PGLYRP1,PGLYRP2,PTAFR,PTPN1,SFTPD,SMAD4,SOCS3,STAT1,TLR4,TLR9,TP63,TRAF6
		Quantity of leukocytes	16.65	ABL1,CAMP,CCR3,CD1D,CD40,CFH,CHUK,CRP,CXCL14,CXCR4,FADD,IFNB1,IL10,IL11,IL12RB2,IL1R1,IL36A,IL36RN,IRAK1,IRF5,LTB,MNT,MR1,NOS2,PGLYRP1,PTAFR,RAD52,RUNX2,SFTPD,SOCS3,SRC,STAT1,TLR4,TLR9,TRAF6
		Inflammatory response	15.93	CAMP,CCR3,CD1D,CD40,CFH,CHUK,CRP,CXCR4,IFNB1,IL10,IL11,IL1R1,IL1RL2,IL36A,IRAK1,LBP,LTF,MCPH1,NOS2,PGLYRP1,PGLYRP2,PTAFR,SFTPD,SOCS3,SRC,STAT1,TLR1,TLR4,TLR9
		Quantity of mononuclear leukocytes	15.75	ABL1,CAMP,CCR3,CD1D,CD40,CHUK,CRP,CXCL14,CXCR4,FADD,IFNB1,IL10,IL11,IL12RB2,IL1R1,IL36A,IRAK1,IRF5,LTB,MNT,MR1,NOS2,PGLYRP1,PTAFR,RUNX2,SFTPD,SOCS3,STAT1,TLR4,TLR9,TRAF6
		T cell development	15.53	ABL1,BRCA1,CD1D,CD40,CHUK,CRP,CXCR4,FADD,IFNB1,IL10,IL12RB2,IL1R1,IL1RL2,IL36B,IRAK1,IRF5,LTB,MNT,PGLYRP2,RAD52,RUNX2,SOCS3,STAT1,TLR4,TP63,TRAF6
		Haematopoiesis of mononuclear leukocytes	15.49	ABL1,BRCA1,CAMP,CD1D,CD40,CHUK,CRP,CXCR4,FADD,IFNB1,IL10,IL11,IL12RB2,IL1R1,IL1RL2,IL36B,IRAK1,IRF5,LTB,MNT,PGLYRP2,RAD52,RUNX2,SOCS3,STAT1,TLR4,TLR9,TP63,TRAF6
		Quantity of cells	15.42	ABL1,BRCA1,CAMP,CCR3,CD1D,CD40,CFH,CHUK,CRP,CXCL14,CXCR4,DMBT1,EFNA3,FADD,FGFRL1,HOXA1,IFNB1,IL10,IL11,IL12RB2,IL1R1,IL1RAP,IL36A,IL36RN,IRAK1,IRF5,LTB,MCPH1,MNT,MR1,NLGN1,NOS2,PGLYRP1,PTAFR,RAD52,RUNX2,SFTPD,SMAD4,SOCS3,SRC,STAT1,TLR4,TLR9,TP63,TRAF6

	Activation of cells	14.99	CAMP,CD1D,CD40,CFH,CHUK,CRP,CXCR4,FADD,IFNB1,IL10,IL11,IL12RB2,IL1R1,IL36A,IL36B,IL36G,IL36RN,IRAK1,IRF5,LBP,LTF,MR1,NOS2,PGLYRP2,RUNX2,SMAD4,SRC,STAT1,TLR4,TLR9,TRAF6
	Quantity of T lymphocytes	14.84	ABL1,CAMP,CD1D,CD40,CHUK,CRP,CXCL14,CXCR4,FADD,IFNB1,IL10,IL12RB2,IL1R1,IRAK1,LTB,MNT,MR1,NOS2,PGLYRP1,PTAFR,RUNX2,SOCS3,STAT1,TLR4,TLR9,TRAF6
	Quantity of lymphocytes	14.36	ABL1,CAMP,CD1D,CD40,CHUK,CRP,CXCL14,CXCR4,FADD,IFNB1,IL10,IL11,IL12RB2,IL1R1,IL36A,IRAK1,IRF5,LTB,MNT,MR1,NOS2,PGLYRP1,PTAFR,RUNX2,SOCS3,STAT1,TLR4,TLR9,TRAF6

The first three columns specify endometrial layers and expression of identified miRNA in the UPA group as compared to proliferative and secretory phase groups. The next three columns specify the biological processes identified using IPA in which mRNAs regulated by the identified miRNA participate.

Table S3. Selection of top ten canonical pathways regulated by the miRNA identified in Table1.

		Canonical Pathway IPA	-log(*p*-value)	mRNA Participating in the Selected Canonical Pathways and Regulated by Identified miRNA
Superficial layer Different from proliferative	Up-regulated	Role of Osteoblasts, Osteoclasts and Chondrocytes in Rheumatoid Arthritis	5.75	SFRP4,AKT2,NFATC2
		Role of Macrophages, Fibroblasts and Endothelial Cells in Rheumatoid Arthritis	5.37	SFRP4,AKT2,NFATC2
		IL-4 Signalling	4.23	AKT2,NFATC2
		RANK Signalling in Osteoclasts	4.07	AKT2,NFATC2
		iCOS-iCOSL Signalling in T Helper Cells	3.94	AKT2,NFATC2
		CD28 Signalling in T Helper Cells	3.88	AKT2,NFATC2
		PI3K Signalling in B Lymphocytes	3.85	AKT2,NFATC2
		Gαq Signalling	3.67	AKT2,NFATC2
		Wnt/β-catenin Signalling	3.62	SFRP4,AKT2
		Role of NFAT in Regulation of the Immune Response	3.58	AKT2,NFATC2
	Down-regulated	Cell Cycle: G1/S Checkpoint Regulation	11.5	MYC,TP53,E2F6,HDAC4,HDAC1,CDK6,CDK4,E2F5,GSK3B,E2F3,CCND1,CDK2,HDAC5
		Aryl Hydrocarbon Receptor Signalling	11.3	TP53,TRIP11,RELA,NFIC,MED1,CDK6,CDK4,RARG,CCND1,CYP1B1,ALDH9A1,AHRR,MYC,SP1,ESR1,CDK2,AHR
		Molecular Mechanisms of Cancer	9.77	RELA,CDK4,ARHGEF1,SMAD5,E2F3,CCND1,BCL2,PTK2,MYC,E2F6,RHOG,E2F5,PRKCE,GSK3B,MAP2K1,PRKD1,ITGB1,TP53,CDK6,GNAI3,MAPK14,NOTCH1,CDK2,WNT1,CTNND1
		p53 Signalling	9.56	TP53,SNAI2,MED1,HDAC1,CDK4,CCND1,PTEN,BCL2,MAPK14,SIRT1,GSK3B,TRIM29,CDK2,DRAM1
		Cyclins and Cell Cycle Regulation	9.17	TP53,E2F6,HDAC4,HDAC1,CDK6,CDK4,E2F5,GSK3B,E2F3,CCND1,CDK2,HDAC5
		Pancreatic Adenocarcinoma Signalling	8.99	TP53,RELA,CDK4,STAT3,E2F3,CCND1,BCL2,PGF,VEGFA,E2F6,E2F5,NOTCH1,MAP2K1,CDK2
		Regulation of the Epithelial-Mesenchymal Transition Pathway	8.93	ETS1,RELA,TWIST2,SNAI2,EGR1,ZEB1,STAT3,MET,DVL2,NOTCH2,ARAF,ZEB2,GSK3B,JAG1,NOTCH1,MAP2K1,WNT1
		Estrogen-mediated S-phase Entry	8.8	MYC,E2F6,CDK4,E2F5,E2F3,ESR1,CCND1,CDK2
		Chronic Myeloid Leukaemia Signalling	8.62	MYC,TP53,RELA,E2F6,HDAC4,HDAC1,CDK6,CDK4,E2F5,

				E2F3,CCND1,MAP2K1,HDAC5
		HGF Signalling	8.27	ETS1,ITGB1,STAT3,CCND1,PTK2,MET,ELF4,PRKCE,MAP3K8,MAP2K1,ELK3,CDK2,PRKD1
Different from secretory	Up-regulated	Neuregulin Signalling	7.56	AKT2,ERBIN,PLCG1,ERRFI1,PTEN
		D-myo-inositol-5-phosphate Metabolism	6.28	PTPN13,PLCG1,PPM1F,PTPN12,PTEN
		Superpathway of Inositol Phosphate Compounds	5.46	PTPN13,PLCG1,PPM1F,PTPN12,PTEN
		iCOS-iCOSL Signalling in T Helper Cells	5.19	AKT2,PLCG1,NFATC2,PTEN
		PI3K Signalling in B Lymphocytes	5.02	AKT2,PLCG1,NFATC2,PTEN
		D-myo-inositol (1,4,5,6)-Tetrakisphosphate Biosynthesis	4.91	PTPN13,PPM1F,PTPN12,PTEN
		D-myo-inositol (3,4,5,6)-tetrakisphosphate Biosynthesis	4.91	PTPN13,PPM1F,PTPN12,PTEN
		3-phosphoinositide Degradation	4.74	PTPN13,PPM1F,PTPN12,PTEN
		Protein Kinase A Signalling	4.39	PTPN13,PLCG1,NFATC2,PTPN12,PTEN
		3-phosphoinositide Biosynthesis	4.32	PTPN13,PPM1F,PTPN12,PTEN
	Down-regulated	Role of Osteoblasts, Osteoclasts and Chondrocytes in Rheumatoid Arthritis	4.73	SRC,JUN,RUNX2,PPP3R1,ITGA2,SMAD4,PPP3CA,WNT5A,IL11
		Guanosine Nucleotides Degradation III	4.24	NT5C3A,NT5E,PNP
		Urate Biosynthesis/Inosine 5'-phosphate Degradation	4.13	NT5C3A,NT5E,PNP
		DNA Double-Strand Break Repair by Homologous Recombination	4.02	ATRX,RAD52,ABL1
		Adenosine Nucleotides Degradation II	4.02	NT5C3A,NT5E,PNP
		Wnt/β-catenin Signalling	3.92	TP53,SRC,JUN,PTPA,ACVR1,ACVR1B,WNT5A
		Purine Nucleotides Degradation II (Aerobic)	3.76	NT5C3A,NT5E,PNP
		Clathrin-mediated Endocytosis Signalling	3.63	MET,SRC,AP2M1,AP2A1,PPP3R1,F2,PPP3CA
		TGF-β Signalling	3.62	JUN,RUNX2,ACVR1,SMAD4,ACVR1B
		Virus Entry via Endocytic Pathways	3.35	SRC,AP2M1,AP2A1,ITGA2,ABL1
Basal layer<br>Different from proliferative	Down-regulated	Aryl Hydrocarbon Receptor Signalling	14.50	TP53,TRIP11,RELA,NFIC,MED1,CDK6,CDK4,RARG,CCND1,CYP1B1,ALDH9A1,CHEK1,MYC,AHRR,CCNE1,HSP90B1,JUN,SP1,CCND3,MAPK3,NFIA,GSTM4,AHR,CDK2
		Pancreatic Adenocarcinoma Signalling	13.70	MAP2K4,TP53,RELA,RAF1,GRB2,FGFR1,CDK4,STAT3,E2F3,CCND1,BCL2,PGF,VEGFA,HMOX1,CCNE1,MAPK3,E2F5,PTGS2,NOTCH1,MAP2K1,CDK2,EGFR
		Molecular Mechanisms of Cancer	13.70	MAP2K4,RELA,RAF1,CDK4,ARHGEF1,SMAD5,E2F3,CCND1,CHEK1,BCL2,MYC,LAMTOR3,RHOG,JUN,CCND3,SUFU,RHOT1,MAPK3,E2F5,PRKCE,GSK3B,MAP2K1,PRKD1,CDC25A,ITGB1,TP53,GRB2,FGFR1,ITGA2,CDK6,GNAI3,CCNE1,MAPK14,WNT3A,NOTCH1,WNT1,CDK2,CTNND1
		p53 Signalling	12.50	TP53,WT1,SNAI2,GRB2,MED1,FGFR1,HDAC1,CDK4,CCND1,CHEK1,BCL2,PTEN,SERPINE2,JUN,MAPK14,SIRT1,GSK3B,TRIM29,CDK2,DRAM1
		Regulation of the Epithelial-Mesenchymal Transition Pathway	12.00	MAP2K4,RELA,RAF1,TWIST2,SNAI2,FGF2,GRB2,EGR1,FGFR1,ZEB1,STAT3,MET,NOTCH2,DVL2,WNT3A,ARAF,MAPK3,ZEB2,GSK3B,JAG1,FGF7,NOTCH1,MAP2K1,WNT1,EG

				FR
		Glioblastoma Multiforme Signalling	11.80	TP53,RAF1,GRB2,FGFR1,CDK6,CDK4,E2F3,CCND1,PTEN,MYC,CCNE1,WNT3A,RHOG,IGF1,RHOT1,MAPK3,IGF1R,E2F5,GSK3B,MAP2K1,CDK2,WNT1,EGFR
		Cell Cycle: G1/S Checkpoint Regulation	11.10	TP53,HDAC4,HDAC1,CDK6,CDK4,E2F3,CCND1,HDAC5,MYC,CCNE1,CCND3,E2F5,GSK3B,CDK2,CDC25A
		IL-8 Signalling	11.00	MAP2K4,RELA,RAF1,GRB2,FGFR1,IQGAP1,CCND1,BCL2,GNG10,PGF,VEGFA,HMOX1,GNAI3,RHOG,JUN,ARAF,CCND3,RHOT1,MAPK3,PRKCE,PTGS2,MAP2K1,PRKD1,EGFR
		Cyclins and Cell Cycle Regulation	10.90	TP53,RAF1,HDAC4,WEE1,HDAC1,CDK6,CDK4,E2F3,CCND1,HDAC5,CCNE1,CCND3,E2F5,GSK3B,CDK2,CDC25A
		HGF Signalling	10.20	MAP2K4,ITGB1,RAF1,GRB2,FGFR1,ITGA2,STAT3,CCND1,MET,ELF4,JUN,MAPK3,PRKCE,PTGS2,ELK3,MAP2K1,PRKD1,CDK2
Different from secretory	Up-regulated	Glucocorticoid Receptor Signalling	4.6	IKBKB,AR,ANXA1,KRT5
		Role of IL-17A in Psoriasis	2.33	S100A9
		TNFR2 Signalling	1.75	IKBKB
		4-1BB Signalling in T Lymphocytes	1.74	IKBKB
		IL-17A Signalling in Fibroblasts	1.73	IKBKB
		Role of RIG1-like Receptors in Antiviral Innate Immunity	1.71	IKBKB
		TWEAK Signalling	1.69	IKBKB
		April Mediated Signalling	1.64	IKBKB
		Role of PKR in Interferon Induction and Antiviral Response	1.63	IKBKB
		B Cell Activating Factor Signalling	1.62	IKBKB
	Down-regulated	IL-10 Signalling	18.8	TRAF6,SOCS3,IL36G,IL1RL2,IL1RAPL2,IL10,IL36RN,IL36A,IL1R1,IL1F10,LBP,CHUK,IL1RAP,IL36B
		Role of Macrophages, Fibroblasts and Endothelial Cells in Rheumatoid Arthritis	17.7	SRC,SOCS3,IL1RAPL2,IL10,IL36A,LTB,IL1F10,IL1R1,TLR9,IRAK1,IL36B,TRAF6,TLR4,IL36G,IL1RL2,IL36RN,TLR1,CHUK,NOS2,IL1RAP,IRAK2
		Toll-like Receptor Signalling	16.5	TRAF6,TLR4,IL36G,IL36RN,TLR1,IL36A,IL1F10,LBP,CHUK,TLR9,IRAK2,IL36B,IRAK1
		Hepatic Cholestasis	16.3	IL1RAPL2,IL36A,IL1F10,IL1R1,IRAK1,IL36B,TRAF6,TLR4,IL36G,IL1RL2,IL36RN,CHUK,LBP,IL1RAP,IRAK2,IL11
		IL-6 Signalling	16.2	TRAF6,SOCS3,IL36G,IL1RL2,IL1RAPL2,IL36RN,CRP,IL36A,IL1R1,IL1F10,LBP,CHUK,TLR9,IL1RAP,IL36B
		Dendritic Cell Maturation	15.6	IL10,IFNB1,IL36A,LTB,IL1F10,TLR9,IL36B,TRAF6,TLR4,CD1D,IL36G,CD40,IL1RL2,IL36RN,CHUK,STAT1
		p38 MAPK Signalling	15.3	IL1RAPL2,IL36A,IL1F10,IL1R1,IRAK1,IL36B,TRAF6,FADD,IL36G,IL1RL2,IL36RN,STAT1,IL1RAP,IRAK2
		Role of Osteoblasts, Osteoclasts and Chondrocytes in Rheumatoid Arthritis	14.8	SRC,IL1RAPL2,IL10,IL36A,IL1F10,IL1R1,TLR9,IL36B,TRAF6,IL36G,IL1RL2,RUNX2,IL36RN,SMAD4,CHUK,IL1RAP,IL11
		Altered T Cell and B Cell Signalling in Rheumatoid Arthritis	14.1	TLR4,IL36G,CD40,IL10,IL36RN,TLR1,IL36A,LTB,IL1F10,CHUK,TLR9,IL36B
		NF-κB Signalling	14.1	IL36A,IL1F10,IL1R1,TLR9,IRAK1,IL36B,TRAF6,FADD,TLR4,IL36G,CD40,IL36RN,TLR1,CHUK,FGFRL1

The first three columns specify endometrial layers and expression state of identified miRNA as compared to the proliferative and secretory phase. The next three columns specify the canonical pathways identified using IPA in which participate the mRNA regulated by the identified miRNA.
